# Cinematic rendering of osteopoikilosis

**DOI:** 10.1093/rheumatology/keae234

**Published:** 2024-04-22

**Authors:** Liwei Fu, Chong Tian, Xianchun Zeng

**Affiliations:** Graduate School of Zunyi Medical University, Zunyi, China; Department of Medical Imaging, Guizhou Provincial People’s Hospital, Guiyang, China; Department of Medical Imaging, Guizhou Provincial People’s Hospital, Guiyang, China; Department of Medical Imaging, Guizhou Provincial People’s Hospital, Guiyang, China

A 26-year-old man presented to the urology department with ejaculatory dysfunction. The patient reported a history of diabetes, and apart from elevated levels of plasma glucose and glycated haemoglobin in the laboratory tests, no other abnormalities were observed. Pelvic CT revealed diffuse dense shadows in the pelvic region and bilateral proximal femurs (Fig. 1A), which were further highlighted using a cinematic rendering reconstruction technique (Fig. 1B and Supplementary Video, available at *Rheumatology* online. After supplementary genetic testing revealed the presence of a *LEMD3* gene mutation in the patient, the physician made a clinical diagnosis of osteopoikilosis based on the combined assessment of the CT examination results.

**Figure 1.**
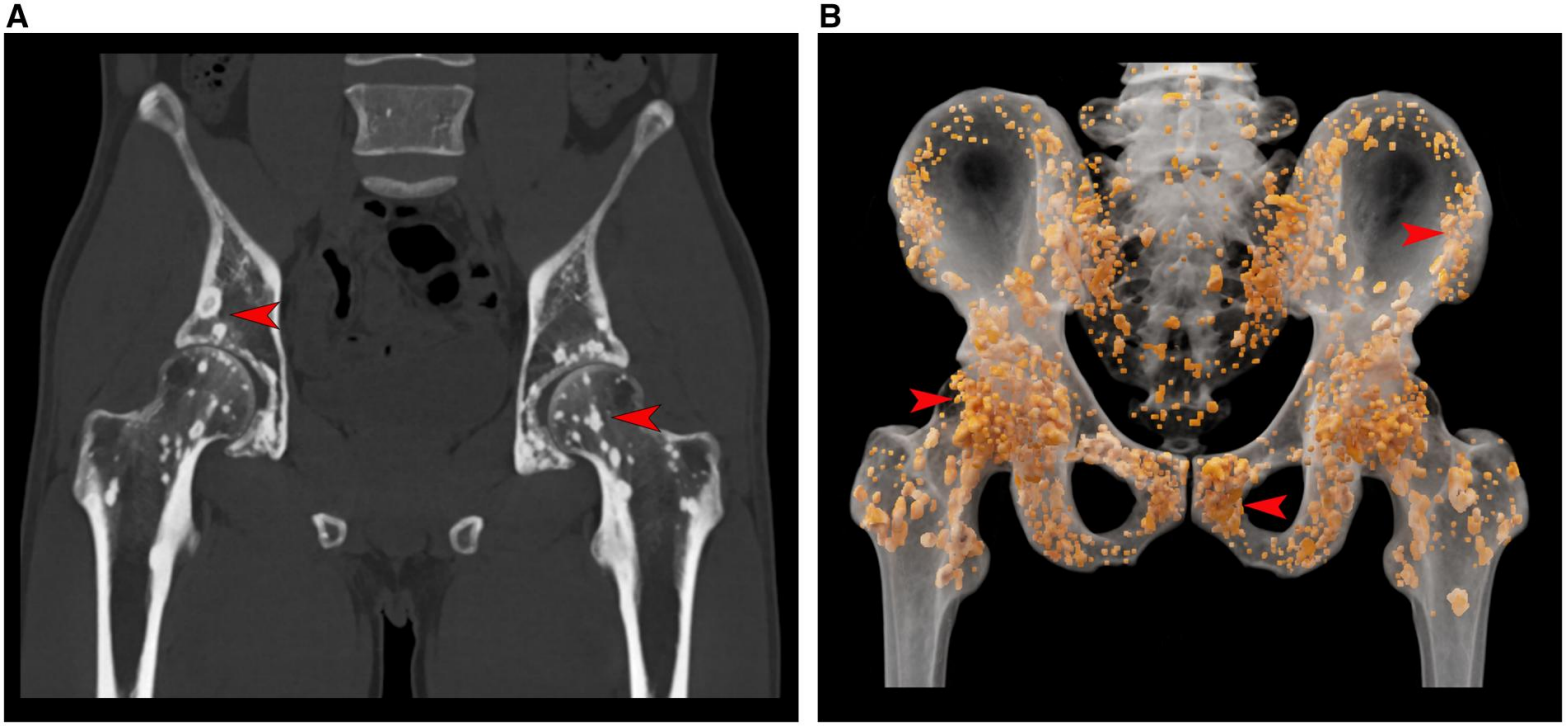
Images from a 26-year-old man with osteopoikilosis. (**A**) Pelvic CT scan in coronal view revealed extensive punctate high-density shadows within the pelvic region and bilateral proximal femurs (arrowheads). (**B**) Coronal cinematic rendering images, rendered individually for each bone lesion, demonstrated osteopoikilosis in 3D (arrowheads)

Osteopoikilosis is a rare asymptomatic osteosclerotic dysplasia, with an incidence of 1 in 50 000 people [1]. Most scholars believe that this condition is associated with mutations in the *LEMD3* gene. These lesions are most commonly discovered incidentally on radiographic imaging, and they are symmetric in nature but with an uneven distribution. Compared with conventional CT images, cinematic rendering can apply different transfer functions for specific cases and structures, aiding more intuitive visualization of spot extent in one view [2].

## Supplementary Material

keae234_Supplementary_Data

## References

[1] Perin S , Rabach I, Pascolo P et al A spotted bone. J Pediatr 2016;176:220–220.e1. 27301574 10.1016/j.jpeds.2016.05.069

[2] Eid M , De Cecco CN, Nance Jr JW, et al Cinematic rendering in CT: a novel, lifelike 3D visualization technique. AJR Am J Roentgenol 2017;209:370–9. 28504564 10.2214/AJR.17.17850

